# Advances in Non-Destructive Testing Methods

**DOI:** 10.3390/ma17030554

**Published:** 2024-01-24

**Authors:** Grzegorz Peruń

**Affiliations:** Department of Road Transport, Faculty of Transport and Aviation Engineering, Silesian University of Technology, 40-019 Katowice, Poland; grzegorz.perun@polsl.pl

## 1. Introduction

Non-destructive testing (NDT) methods are a group of tests allowing one to detect external (surface) as well as internal defects of a structure. They allow one to determine the state of micro- and macro-structures without interfering with the structure of the tested object, providing information about the functional properties of the tested object. This basic feature and the variety of non-destructive testing methods make them applicable in many industries. They are notable in the diagnosis of infrastructure defects and means of air transportation and rail transportation, but are also applicable in the automotive industry [1,2,3]. The most obvious field of application of NDT is the quality control of final products, as well as the verification of materials and semi-finished products used in manufacturing processes. Theoretically, non-destructive testing, unlike destructive testing, can be applied to all manufactured pieces of a product; moreover, these methods are used during operation to assess the technical conditions of objects. A limitation of the number of tests conducted may be the cost of implementation and the time required to conduct the tests [4,5]. Despite these numerous advantages, NDT methods also have limitations, where one of which is the economic factor of the cost of purchasing testing equipment [6]. NDT methods can be carried out at various stages of production, operation, or repair. They are used to carry out quality control in production and to determine the technical conditions of an object, respectively. With NDT, it is also possible to detect operational defects, including corrosion, erosion, or cracks.

Unlike the well-known destructive testing methods that have been in use for a long time, a large number of testing methods classified as non-destructive have been in use relatively recently. At present, a wide variety of non-destructive testing methods, operating on the basis of different phenomena, can be specified. A detailed classification and description can be found in the work of [7,8,9] and in the EN-ISO/ISO standards, among others. The most common non-destructive tests include the following:

- Visual methods;
- Penetrant methods;
- Magnetic powder methods;
- Methods using eddy currents;
- Thermographic methods;
- Vibration methods;
- Acoustic methods;
- Ultrasonic methods;
- Radiographic methods.

Visual, penetrant, and electromagnetic methods, including methods using eddy currents, are ideal for detecting surface discontinuities. The detection of subsurface defects is made possible by eddy current, magnetic particle, and thermographic methods. Among the methods of non-destructive volumetric testing, acoustic, ultrasonic, and radiographic methods stand out.

## 2. A Statistical Look at the Research Presented in this Topic

**Figure 1:**
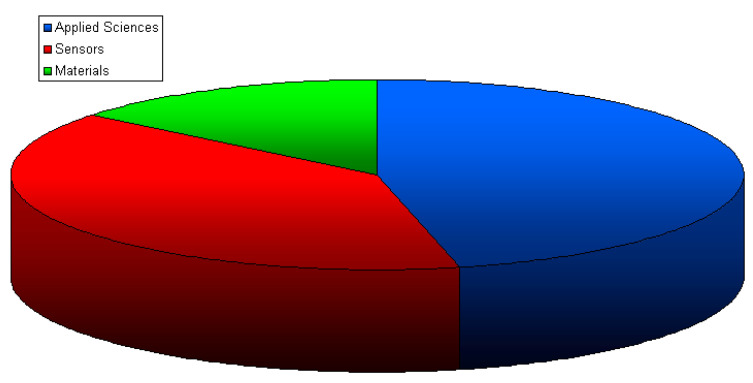
Participation of individual journals in the topic “Advances in Non-Destructive Testing Methods”.

The purpose of this Topic was to present both the development of various non-destructive testing methods and examples of applications of this group of tests in various industries. Thanks to the fact that the scope of this topic was not strictly limited to defined methods, this study includes the presentation of a very wide spectrum of research and applied methods. The presented research was carried out by research centers located all over the world, and a brief description of selected studies is presented in the next chapter. Thirteen papers were published in *Applied Sciences*, eleven were published in *Sensors*, and four were published in *Materials*. The published papers include 142 authors from many institutions. These simple statistics, presented in Figure 1, show that the topic has received considerable interest and thus confirm the universality and popularity of NDT research.

## 3. Summary of the Contributions

In one article [10], the authors propose a convolutional-neural-network-model-based through-focus scanning optical microscopy (TSOM) method for measuring individual high-aspect-ratio (HAR) grooves on silicon up to 30 µm wide and 440 µm deep. The standard deviation and error were about one hundred nanometers or less in value.

Grazing-incidence small-angle X-ray scattering (GISAXS) is an effective method for the structural analysis of ordered arrays of quantum dots, nanoparticles, or similar objects. In [11], the authors demonstrate a software platform to analyze and simulate 2D GISAXS intensity distributions from ordered networks of various nanoparticles.

The possibilities of using non-destructive testing methods in transportation are presented in [12] using air transportation as an example. High-energy radiation sources are used to provide strong safety inspection capabilities using a non-invasive imaging system. The authors present an air cargo inspection system using multiple radiation sources, a D-T neutron generator, and an accelerator-based gamma-ray source to classify plastics, metals, and organics among various sample materials. Radiation can penetrate materials tens of centimeters thick in an air cargo container.

Similarly, aerospace transportation is an area of interest for the authors of [13]. The purpose of this paper is to conduct a reliable non-destructive evaluation (NDE) of glued aerospace components by developing new multidimensional data fusion techniques that combine information obtained using ultrasonic and X-ray NDE methods.

NDT methods are also being applied to marine transportation. The marine composite market is driven by growing demand for lightweight, corrosion-resistant, and impact-resistant boats. Polymer matrix composites are currently the most popular composite materials in marine applications. Ref. [14] presents a numerical analysis of the feasibility of using vibrothermography to detect defects in fiberglass-reinforced laminates in marine applications.

Pipelines are also related to transportation, which are also of interest to those involved in non-destructive testing. Problems related to the classification and identification of defects in polyethylene (PE) gas pipelines are presented in reference [15]. The paper proposes a framework for classifying and identifying defects of underground PE gas transmission pipes. The authors prove the possibility of fast and accurate detection of defects in PE pipelines.

Ref. [16] presents a less expensive and simpler way to apply the time delay estimation method to locate leaks in underground pipelines. The accuracy of the TDE depends on the acoustic velocity and attenuation of the leak signal propagating along the pipeline. The analytical predictive model is the typical approach for obtaining the propagation velocity and attenuation of leakage waves. To determine the effective design variables in the algorithm based on the embedment parameters of the pipe, this study analyzed the sensitivity of soil parameters to the wave number.

Three-dimensional localization of buried polyethylene pipes is a problem addressed in the work of [17]. The authors developed a multi-sensor method based on the principle of pipe vibration, which allows estimating the depth, is non-invasive and non-destructive, and does not require prior information about the propagation medium.

In order to solve the problem of quantifying magnetic flux leakage (MFL) detection signals damaged in the operation of oil and gas pipelines, an inhomogeneous magnetic charge model based on magnetic effects was developed. Reference [18] discusses the force–magnet coupling relationship in the inhomogeneous magnetic charge model, develops a theoretical model of complex magnetic charge, and analyzes the pattern of changes in the characteristic values of MFL signals under elastic stress and plastic deformation.

In reference [19], the authors address the problems associated with measuring the moisture content of building materials. This is important from the point of view of both the process of building construction and the subsequent operation of said buildings. In engineering practice, indirect moisture measurement techniques, mainly resistive and capacitive, are the most popular. The main objective of this research work was to compare the classical TDR measurement technique with non-invasive surface TDR sensors.

Ref. [20] proposes an inversion algorithm for predicting dynamic moduli using CNNs which is trained using samples related to the dynamic response of a multilayer concrete pavement structure obtained using SEM. The proposed method acts as a reliable test tool for the falling weight deflection gauge technique of pavement structures.

The contribution of the study of [21] is the use of broadband ultrasonic attenuation techniques in concrete-filled steel pipe samples to locate air voids. The results show a linear relationship between the average BUA in the window of concrete-filled steel pipes and the percentage of air voids: the higher the percentage of air voids, the higher the BUA values.

The meso-scale failure properties of concrete and mortar were investigated experimentally in [22] via Brazilian disc split tensile tests combining X-ray computed tomography (CT) and digital image correlation (DIC) technology. Taking into account water/cement ratios and loading rates, the effects of meso-components on the macro tensile properties and failure modes of concrete were studied.

In reference [23], the longitudinal nonlinear ultrasonic parameters of carbonatized concrete were detected using an embedded composite piezoelectric transducer (ECPT) as a transducer, and the effect of temperature on the nonlinear parameters of carbonatized concrete was investigated. Higher variation in nonlinear parameters at the same time of carbonation was observed for samples with a high water–cement ratio than for samples with a low water–cement ratio.

The purpose of the work of [24] was to develop a technique for measuring the thickness of non-conductive coatings on non-magnetic conductive base metals. The authors used the amplitude-phase eddy current method and algorithms for processing measurement information, taking into account the effect of the base metals’ specific electrical conductivity. They found two-dimensional thickness gauge graduation characteristics using several base metals with different values of specific electrical conductivity.

An innovative analytical protocol based on external reflection FTIR spectroscopy (ER-FTIR), combined with spectral deconvolution and multivariate data analysis, was used in [25]. The ER-FTIR technique is fast, portable, and widely used in the field of cultural heritage, but rarely applied to the study of textiles. This is the first time the ER-FTIR band assignment for silk has been discussed.

A completely different topic was addressed in reference [26]. A tracking model called Road-TransTrack based on transformer optimization was proposed to track and count the number of damages in road videos. The tracking and counting model was improved with a transformer and a self-observation mechanism to improve the accuracy of damage detection and counting in road videos.

The research topic of [27] is the generation of high-quality phase-contrast computed tomography images with given incomplete projections. The authors present the potential for expanding applications of PCCT techniques in the fields of composite and biomedical imaging and describe a two-domain (i.e., projection sinogram domain and image domain) deep-learning-based enhancement framework for PCCT with sparse projections. It consists of two convolutional neural networks (CNNs) in two domains and a phase-contrast Radon inversion layer (PCRIL) to connect them.

Ref. [28] presents an approach to efficiently detect local defect resonances in solids with localized defects and a method for determining the frequency of local defect resonances. The algorithm is based on the observation that vibration amplitudes are significantly higher for the modal shape of a local defect than the average value for a whole structure.

The work of [29] investigates surface roughness measurements using piezoelectric acoustic discs and corresponding signal processing. Surface roughness is one of the characteristics of surface texture, which can have various irregularities related to manufacturing methods. The study concludes that acoustic friction measurement shows promising results as a new method for measuring the surface roughness states of some materials.

In [30], the authors study various contact states of a high-voltage SF6 circuit breaker considering different currents in power system applications. This paper aims to present a novel method for detecting the state of HVCB contacts based on a vibration signal. The experimental and theoretical analyses used can provide a reference for future fault monitoring and diagnosis in HVCBs.
